# 2180. *Pseudomonas aeruginosa* epidemic high-risk clones and their association with multidrug-resistant

**DOI:** 10.1093/ofid/ofad500.1802

**Published:** 2023-11-27

**Authors:** Jeannete Zurita, Gabriela Sevillano, Maria Belen Solis, Ariane Paz y Miño, Beatriz Rizkallah Alves

**Affiliations:** Unidad de Investigaciones en Biomedicina. Zurita & Zurita Laboratorios, Quito, Pichincha, Ecuador; Unidad de Investigaciones en Biomedicina. Zurita & Zurita Laboratorios, Quito, Pichincha, Ecuador; Unidad de Investigaciones en Biomedicina. Zurita & Zurita Laboratorios, Quito, Pichincha, Ecuador; Mass General Brigham Salem Hospital, Salem, Massachusetts; Mass General Brigham Salem Hospital, Salem, Massachusetts

## Abstract

**Background:**

*Pseudomonas aeruginosa* (*PAE*) is an opportunistic pathogen associated with a variety of hospital infections. Treatment of infections caused by *PAE* is challenging due to the emergence of multidrug-resistant (MDR), which is associated with increased morbidity, mortality, and healthcare costs. Clonal spread and the presence of resistance genes probably explain the successful propagation of MDR. In Ecuador, data on the molecular epidemiology, as well as circulating clones, are limited. Therefore, the objective of this study is to know the population structure of *PAE* by identifying clones in clinical samples in Quito-Ecuador.

**Methods:**

A significant set (45) of randomly selected clinical *PAE* isolates, including multidrug and non-multidrug resistant isolates, were assigned to sequence types (STs) and compared them with their antibiotic susceptibility profile. The genetic diversity was assessed by applying the multilocus sequence typing (MLST) scheme (Figure 1) and the genetic relationships between different STs were corroborated by the phylogenetic neighbor-net network (Figure 2).

**Figure 1.**
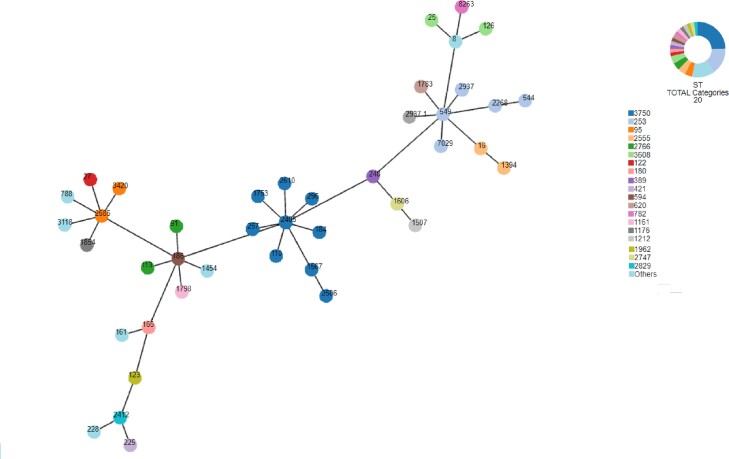
Analysis of the allelic profiles using eBURST algorithm.

**Figure 2.**
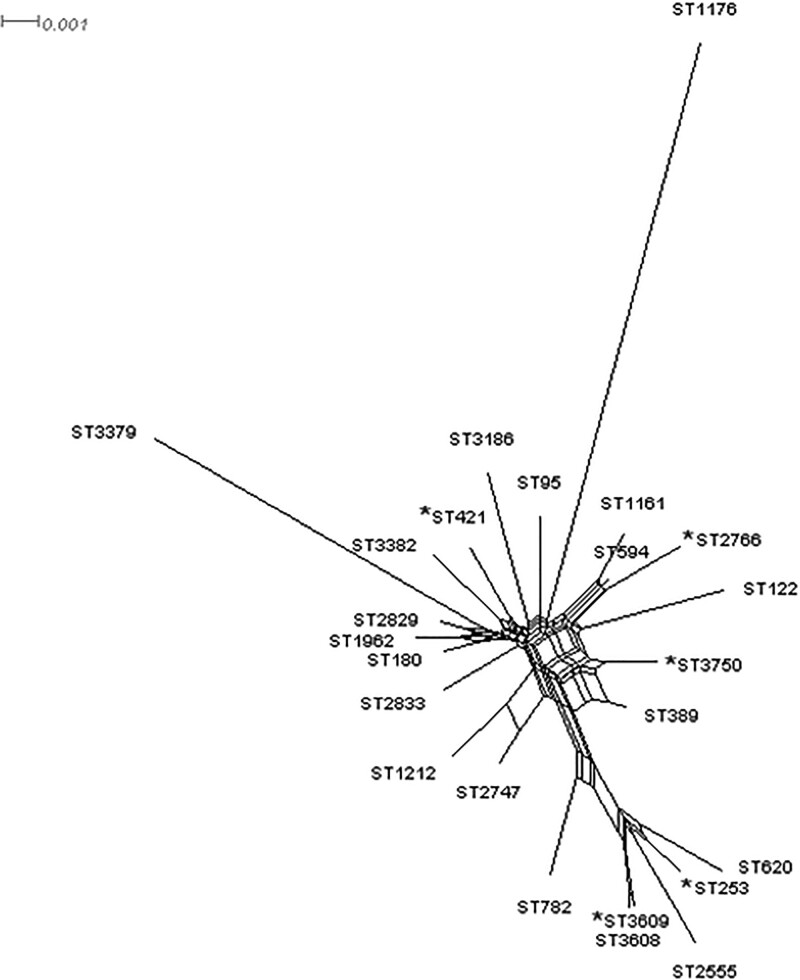
Splits Tree showing the distribution of all the sequence types obtained for the clinical Pseudomonas aeruginosa isolates studied. The Splits Tree was based on the analysis of the allelic profiles of the acsA, aroE, guaA, mutL, nuoD, ppsA and trpE genes. Asterisk mark (*) indicates the MDR isolates

**Results:**

The analysis of seven loci, acsA, aroE, guaA, mutL, nuoD, ppsA and trpE, identified 24 different ST and the most prevalent STs were ST-3750 and ST-253. A SplitsTree was constructed with all the isolates analyzed. The majority of the MDR isolates were included in ST-3750 and ST-253, also 3 singleton ST was identified in to MDR isolates. The 20 different ST was found to non-MDR isolates, and only 3 ST were found in more the one isolates.

**Conclusion:**

The population structure of clinical *PAE* presents in these isolates indicate a significant association between MDR isolates and the clonal types: all ST-3750 and ST-253 isolates were MDR. ST-3750 is closely related strains to the ST111 (CC111), and ST-253 and ST111 are a group of successful high-risk clones and widely distributed worldwide. The multiresistant-isolates studied are grouped in the most prevalent sequence types found, and the susceptible isolates correspond mainly to singleton sequence types. Therefore, these high-risk clones and their association with multidrug resistance phenotypes are contributing to the spread of MDR in Quito-Ecuador.

**Disclosures:**

**Jeannete Zurita, n/a**, Pfizer: Grant/Research Support

